# A tool for immediate and automated assessment of resuscitation skills for a full-scale simulator

**DOI:** 10.1186/1756-0500-4-550

**Published:** 2011-12-20

**Authors:** Christian M Schulz, Valentin Mayer, Matthias Kreuzer, Eberhard F Kochs, Gerhard Schneider

**Affiliations:** 1 Department of Anaesthesiology, Klinikum rechts der Isar, Technische Universität München, Ismaninger Str. 22, 81675 Munich; 2 Department of Anaesthesiology, Rotkreuzklinik München, Nymphenburger Str. 163, 80634 München; 3 Department of Anaesthesiology, Klinikum rechts der Isar, Technische Universität München, Ismaninger Str. 22, 81675 Munich; 4 Department of Anaesthesiology, Klinikum rechts der Isar, Technische Universität München, Ismaninger Str. 22, 81675 Munich; 5 Department of Anaesthesiology I, University Witten/Herdecke, Helios Klinikum Wuppertal, Germany

## Abstract

**Background:**

For performance assessment during simulation, mostly observers rate the trainees' performance using checklists. Simulator outcome may provide immediate and objective feedback to the participants but requires additional work for the accurate scenario design. High-fidelity simulators are based on physiologic models and store all changes of the simulator conditions during the scenarios and may therefore be used for the assessment of performance. In the present work, the design of a simulator script for the assessment of resuscitation skills using an Emergency Care Simulator (ECS, METI, Sarasota, Florida) is described.

**Findings:**

A standardized resuscitation simulator script and a visual basic-based macro were programmed for the immediate and automated extraction of performance-related variables from the log files. The following parameters were assessed: mean cardiac output, time until return of spontaneous circulation, no-flow-time, no-flow-time fraction, the time until the first defibrillation, the number and fraction of indicated and non-indicated defibrillations. Furthermore, mean deviation of defibrillation interval from the 2 minutes interval, the mean interval of defibrillations and the time until the first administration of epinephrine were calculated. As an example, the results of resuscitation efforts according to 2005 guidelines by five teams that consisted of one emergency physician and two paramedics are presented. No data are provided about its validity and reliability.

**Conclusion:**

The tool can be used to assess adherence to European and American cardiopulmonary resuscitation guidelines (both 2005 and 2010) and to compare simulator outcome if different guidelines are trained and applied according to specific curricula. It represents an example of how simulator outcome can be used for performance assessment and may help to design more complex test-scenarios including the field of critical incidents in anesthesia.

## Background

The development of appropriate scenario designs for the assessment of performance remains challenging in simulator settings. So far, in most studies performance is assessed by observer ratings [1-5] or questionnaires [6,7]. The use of simulation technology results in increased skills of residents during cardiopulmonary resuscitation (CPR) scenarios [5,8-12]. Simulator outcome-based performance measures may provide an efficient and immediate feedback to the participants but require additional work on scenario design and are not implemented in most settings [1]. Once established, they are easy to apply, can provide results immediately and are observer-independent.

Recently, high-fidelity simulation has been introduced in CPR training [13]. With the aim to provide feedback in real time and a comprehensive learning environment, most high-fidelity simulators use physiological models that allow realistic and dynamic behaviour on trainees' actions. These models integrate a large variety of physiologic parameters mainly of the cardiovascular (e.g. right ventricular contractility or systemic vessel resistance) and the pulmonary system (e.g. chest wall compliance, shunt fraction). These variables can be modified either by a priori programmed scripts, by the simulator technician or by the automatic detection of trainees' actions. Administered drugs also influence the model according to their pharmacodynamic and pharmacokinetic profile. For example, the administration of propofol results in a lower blood pressure, apnoea and the simulator may close the eyes. As long as no ventilation is recognized, the oxygen partial pressure will decrease and as a result, the measured value saturation will deteriorate. Thus, high-fidelity simulators were developed to train complex and dynamic incidents in anaesthesia and emergency medicine. The new guidelines emphasize the importance to prematurely identify the patients that suffer from a high risk of sudden cardiac arrest [14]. Such constellations may be trained better with high-fidelity simulation than on technically more simple cardiac arrest mannequins.

The Emergency Care Simulator (ECS, Meti^®^, Sarasota, Florida, US) is such a high-fidelity human patient simulator driven by a physiologic model. It automatically senses chest compressions, mechanical ventilation and defibrillations. During each scenario, the simulator software records three different files that store events (event log), physiologic data (physio log) and administered drugs (drug log). Scripts can be programmed with the aim to present standardized scenario contents and to reduce control complexity. These scripts usually consist of different states that define simulator conditions and may be combined with multiple simulator actions and/or reactions. A physician (or technician) drives the simulator with or without scripts and has to observe attentively the trainees and their actions during the scenario.

So far, an automated assessment of skills during resuscitation is available for other low-fidelity mannequins [15,16] but not for the ECS. With the aim to provide rater-independent and immediate feedback about the trainees' performance to the trainers during cardiopulmonary resuscitation training, we developed a tool that uses a standardized script requiring minimal interventions by the observer. The present paper describes the development of a dynamic cardiopulmonary resuscitation scenario, the extraction of relevant data of the log files and finally presents the results obtained by 5 teams that consisted of one emergency physician and two paramedics.

## Implementation

### Development of the CPR-script

**Figure 1:**
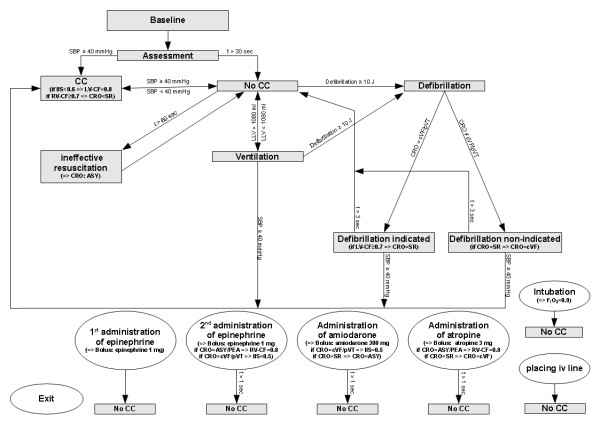
**The configuration of the simulator script**. The angular grey boxes represent simulator states that are passed through automatically according to the fulfilling of the conditions (arrows). The round white boxes are the states that have to be marked by the observer ("marker state") as soon as their content is performed by the trainees. After each marker state, the simulator returns into the state "no chest compressions". The state "exit" permits full simulator control to the observer immediately. Abbreviations: ASY = asystole, CC = chest compression, CF-LV = contractility factor left ventricle, CF-RV = contractility factor right ventricle, CRO = cardiac rhythm override, cVF = coarse ventricular fibrillation, IIS, ischemic index sensitivity, LLV = left lung volume, pVT = pulseless ventricular tachycardia, SBP = systolic blood pressure, SR = sinus rhythm, t = time in state

With the aim to provide a standardized and realistic scenario content reflecting resuscitation guidelines of the year 2005, we decided to develop a script (Additional file 1: CPRscript.hs6) that dynamically reacts to the trainees' actions (figure 1), for the initial situations asystole, pulseless ventricular tachycardia and ventricular fibrillation. The script is subdivided into different states representing specific simulator conditions. The simulator automatically switches between the states "chest compression", "no chest compression", "ventilation", "ineffective resuscitation", "defibrillation", "defibrillation indicated", "defibrillation non-indicated" according to the actions undertaken by the trainees. Defibrillations were automatically recorded as soon as the power was delivered to the two electrodes that were attached on the simulator's thorax at the customary locations. With the aim to enhance safety, the trainees were allowed to use energy levels lower than 200J. All defibrillations performed with more than 10J power that were performed on the electrodes were recorded. According to the heart rhythm at this moment, they were classified either as indicated or non-indicated. Within the states, specific conditions (e.g. if systolic arterial pressure is superior to 40 mmHg then go to state "chest compression") are defined and result in transitions from one to another state. The simulator used for the present study has no means to detect tasks like intubation or placing of a intravenous line automatically and it was not equipped by a barcode-reader-based drug recognition system so that six additional states are used as marker states: "placing intravenous line", "intubation", "first administration of adrenaline", "second administration of adrenaline", "administration of atropine" and "administration of amiodarone". When the corresponding action is observed by the trainer, a mouse click on this status results in a time mark that is written to the event log. At this point, the tool is not fully automatic and requires an attentive observer.

Additionally, the marker states "second administration of adrenaline" or "administration of amiodarone" followed by chest compressions change the simulator conditions in such a way that the next correctly performed defibrillations will show therapeutic effect. This is achieved by the simulator variables ischemic index sensitivity (set to 0.5 when the observer clicks the marker states "second administration of adrenaline" or "administration of amiodarone") and the left ventricular contractility factor (set to 0.8 if the ischemic index sensitivity is already 0.5 and chest compressions are performed, 0.8 being the condition for the defibrillations to convert a ventricular fibrillation or a pulseless ventricular tachycardia into sinus). Analogically, the variable right ventricular contractility factor was set to 0.8 for the effect of atropine. This proceeding results in the need for chest compressions after administration of drugs to simulate their transport to the target receptors. When the cardiac rhythm was a pulseless ventricular tachycardia or fibrillation and no chest compressions were performed during more than 60 seconds, the simulator condition turned into asystole.

With the aim to guarantee the same starting conditions for each scenario we created three different patients for the following initial cardiac rhythms at the beginning of the scenario: asystole, ventricular fibrillation and pulseless ventricular tachycardia. Each patient is based on the standard "untitled adult patient" provided by the simulator software. For all of them, the variables "fixed neuromuscular blockade" (set to 100%), "fraction of inspired oxygen" (set to 80%), " ischemic index averaging" (set to 0.99), "ischemic index sensitivity" (set to 0.5), "contractility factor left ventricle" and "contractility factor right ventricle" (both set to 0.5) were changed equally as needed to make the script work. All other variables were left at the default values. As soon as the cardiac output (being an integral of blood flow related to time and therefore decreasing as function of time) reached zero, the conditions were saved and represented the initial patient state or the beginnings of the scenario (baseline). In conclusion, the three patients differed only in the variable "cardiac rhythm override" and all had a cardiac output of zero at the beginning of the scenario.

### Analysis of the simulator log files

In a first step, the log files recorded during cardiopulmonary resuscitation were analysed with regard to their usefulness for resuscitation performance assessment. The event log provides information about the cardiac rhythm but does not detect whether cardiac compression is performed, and the moment of intubation is not logged. The physio log registers heart rate, systolic and diastolic blood pressure, central venous pressure, systolic and diastolic pulmonary arterial pressure, pulmonary capillary wedge pressure, cardiac output, tidal volume during spontaneous respiration, spontaneous respiratory rate, alveolar and arterial partial pressure of O2, CO2 and N2 and oxygen saturation. Moreover, it provides data about haemoglobin concentration, blood temperature, partial venous pressure of O2 and CO2 that were considered irrelevant for performance assessment during resuscitation efforts according to the guidelines. The drug log registers time point, type and quantity of the drugs registered by the software. Mechanical ventilation, chest compression depth and frequency interact with the physiologic model but unfortunately their parameters are not recorded in the log files and therefore not available to the user. The simulator's arterial partial pressures of O2 and CO2 may serve as markers for ventilation efficacy but react slowly and require that the observer recognizes whether and how much oxygen is administered. If the simulator is in functional cardiac arrest, no diffusion occurs between alveoles and blood in the physiologic model. Consecutively, the oxygen saturation decreases. At the same time, alveolar partial pressure of oxygen increases even though no ventilation is performed. As soon as chest compressions are recommenced, oxygen saturation and arterial partial pressure of O2 increase without any ventilation having been realized. Unfortunately, this behaviour can not be influenced by the user. We considered this finding unrealistic and therefore, these parameters were excluded from the performance assessment.

### Data extraction from the log file

The resulting log files (file format: txt) were processed in Microsoft Excel using a VisualBasic macro RPMACRO to extract the relevant variables (Additional file 2: RPMACRO_JournalVersion.xls). RPMACRO formats and searches the generated log files to obtain information regarding the overall time until return of spontaneous circulation (ROSC), mean cardiac output, no-flow-time and no-flow-time fraction. Where ever possible, the terminology suggested by Johanson-Kramer was used [17]. The time until the first defibrillation was calculated as well as the number and fraction of indicated and non-indicated defibrillations. As additional information, the mean deviation of defibrillation interval from the 2 minutes interval and the mean interval of defibrillations are displayed in the resulting Excel sheet. The time until a possible first administration of adrenaline is also calculated. As soon as the simulator's trachea was intubated, it was possible to perform chest compressions without interruptions. Therefore, the simulator remained in the state "chest compression". The tool was not able to record ventilation manoeuvres correctly in this case. Accordingly, only the total number of ventilations and their mean frequency for the period before intubation are extracted from the log files. All these parameters are added to the opened Event-log file and saved to an "AfterMacro_Eventfile". This routine executes after clicking on the EVENT - button. If clicking on the PHYSIO button, median cardiac output is calculated from combined information derived from the Event- and Physio-log and added to the Physio-log and saved in the "AfterMacro_Physiofile".

### Data collection

With the aim to get results from experts, five professional teams consisting of one emergency physician and two paramedics were asked to participate in one test scenario. Each team member has regularly worked in prehospital emergency medicine and is thus familiar with cardiopulmonary resuscitation algorithms. No prior simulator training was provided to any team. Before entering the scene, a short introduction about the simulator and the equipment was given. After the scenario, a short debriefing was provided and the teams were asked not to talk about scenario content. The teams were tested independently on different days. Thus, there was no learning by observation. The results from the test scenarios were presented descriptively without performing exploratory data analysis.

## Results

### Test results

The script could be used for all teams without any changes. In every team, the complete outcome data was usable. The results of the 5 teams are presented in table 1. They found a coarse ventricular fibrillation as initial rhythm and performed the resuscitation according to European guidelines of 2005. No-flow-time fraction was between 0.13 and 0.44. The mean interval between defibrillations differed especially between team 1 (3:47 min) and team 5 (2:22 min). Accordingly, time to ROSC was longer in team 1 (18:09 min) than in team 5 (8:52 min). However, team 1 had the lowest no-flow-time fraction. In team 3, a high no-flow-time fraction and a relatively long mean deviation of defibrillation interval from the 2 minute interval, but had the shortest time to ROSC was observed. This occurred because the team intubated the simulator's trachea, placed an intravenous line and administered epinephrine and amiodarone between the first and the third defibrillation and thus the simulator converted to sinus rhythm relatively soon. Accordingly, no-flow-time fraction was relatively high. The calculated frequency of ventilations was very low in all teams.

**Table 1:** Results of 5 prehospital emergency teams consisting of one emergency physician and two paramedics

Parameter	Team 1	Team 2	Team 3	Team 4	Team 5	Mean ± SD
	1	2	3	4	5	
median cardiac output (l * min^-1^)	1.2	1.5	1.4	1.8	1.5	1.5 ± 0.2

no-flow-time fraction	0.21	0.24	0.44	0.13	0.30	0.26 ± 0.10

time until ROSC (min:sec)	18:09	12:08	07:43	12:41	08:52	11:53 ± 03:39

time until first administration of epinephrine (min:sec)	08:56	06:40	05:05	10:10	07:35	07:39 ± 01:52

time until first defibrillation (min:sec)	03:01	00:07	01:44	01:40	01:47	01:38 ± 00:58

indicated defibrillations (n)	5	6	3	5	4	4.6 ± 1.0

non-indicated defibrillations (n)	0	0	0	0	0	0

mean deviation of defibrillation interval from the 2 minutes interval (min:sec)	01:47	00:26	01:08	00:43	00:24	00:54 ± 00:31

mean interval of defibrillations (min:sec)	03:47	02:24	03:06	02:43	02:22	02:52 ± 00:32

overall ventilations before intubation (n)	20	6	10	4	13	10.6

mean frequency of ventilations before intubation (n * min^-1^)	1.1	0.5	1.3	0.3	1.5	0.9 ± 0.5

The initial rhythm was ventricular fibrillations in all scenarios (SD = standard deviation).

## Discussion

The presented tool assesses descriptive variables during simulated CPR using an ECS. The script works with any guidelines. The tool can be used to detect discrepancies between actual performance and trained guidelines and allows immediate and task-specific (e.g. time until adrenaline, interval between defibrillations) feedback during debriefing. The approach of combining CPR outcome variables with the physiologic models of high-fidelity simulation may facilitate targeting of training directly to more relevant physiological and performance outcomes. The physiological feedback may be used to evaluate new technology in addition to guideline quality [18]. Moreover, it may contribute to optimize the team work in the challenging task of cardiopulmonary resuscitation when a lot of tasks have to be performed under pressure of time.

For example in team 3, placing an intravenous line, administration of epinephrine and amiodarone in a short period of time and the following defibrillation resulted in an early ROSC because all the tasks required by the script for an effective defibrillation had been performed. The large quantity of tasks in a short time is reflected by a very high no-flow-time fraction. This finding suggests that the parameter time until ROSC is neither a useful surrogate of guideline adherence nor a good indicator for overall performance. However, other data can be used to determine adherence to guidelines (e.g. time until 1^st ^administration of adrenaline, mean interval of defibrillations and no-flow-time fraction).

The calculated ventilation rate before intubation was very low (mean 0.9 min^-1 ^± 0.3). Thoren reported the percentage of correct inflations as low as 6.5%. Given a rate of 5.4 min^-1 ^in a sample size of 10 professionals, a ventilatory rate of 0.3 min^-1 ^of correct inflations could be calculated^16^. In another observational study that was performed to compare the 2000 and 2005 guidelines, no significant difference in the ventilatory rates (6.5 ± 0.4 vs 5.2 ± 0.6 min^-1^, p = 0.084) was found between the guidelines [19]. In both studies the ventilatory rate was severalfold higher than in the present investigation. Given that our sample size consisted of professionals, this finding suggests that the presented tool requires further refinement with respect to a valid assessment of the ventilation.

Some additional limitations have to be mentioned: So far, no data about validity and reliability of the presented assessment tool are available and require additional investigation by application of additional measures of resuscitation performance or by comparing results of repeated exposure of both experts and novices (1). After repeated exposure to standardized scenarios, the trainees may learn to treat effectively the simulator (but not a real patient) and there is no evidence that an improvement in the real world is achieved (2). The ECS does not provide data about chest compression depth and frequency and comparability to other studies is therefore impaired (3), the criteria for an effective ventilation with regard to tidal volume and frequency are limited and include only the time before intubation. This requires further refinement of the tool (4). The comparability of the cardiac output is impaired due to missing calibration possibility of the chest compression sensor (5). The adherence to specific guidelines is not assessed but can be derived from the results easily. The simulator script, the presetting of the scenarios and the visual basic macro are provided as internet supplements and can be adapted according to the requirements of the users. In the present investigation, the tool was applied in the ECS system. As the driving software (METI HPS 6.4) is identical to the software used by the Human Patient Simulator (HPS^®^, METI, Sarasota, Florida), this tool should be also applicable in the HPS. However, this was not tested. Future developments may combine physiological models and a direct assessment of compression depth and no-flow-time. This would result in an interesting approach to assess a large variety of aspects for CPR performance and enhances the comparability to results of studies on simpler cardiac arrest trainers.

## Conclusions

The presented tool may be of value for trainers and researchers using full-scale simulators of Meti (Sarasota, Florida) who want to give immediate feedback to performance-related variables or who want to use it for testing the effect of different interventions (e.g. different types of training curricula, technical devices, different guidelines). Moreover, it provides an example of how simulator scenarios can be programmed to automatically generate metrics which may be of value in assessing performance in full-scale simulator environments. With the aim to enhance realism of the script, further refinement of the tool may include a no-flow-time dependent time until ROSC and an assessment of ventilation quality after successful intubation.

## Availability and requirements

**Project name**: Resuscitation scenario standardization and performance assessment for ECS simulators

**Project home page**: none

**Operating systems**: CPRscript is based on Mac OS X 10.5.8; RPMACRO is based on Microsoft Windows XP Professional Version 2002 Service Pack 3.

**Programming language**: CPRscript is a batch file in plain text; this batch file is executed by METI HPS 6.4 for HPS software. RPMACRO was written in Microsoft Visual Basic Version 6.5.1053 using Microsoft Excel 2003 (11.8335.8333) Service Pack 3

**Other requirements**: none

**License**: none

**Any restriction to use by non-academics**: none

## Availability of supporting data

The data sets supporting the results of this article are included within the article and its additional files.

## List of abbreviations

CPR: cardiopulmonary resuscitation; ECS: Emergency Care Simulator (METI, Sarasota, Florida); ROSC: return of spontaneous circulation.

## Competing interests

The authors declare that they have no competing interests.

## Authors' contributions

CS was responsible of study design and drafted the manuscript. VM programmed the simulator script tested it and acquired the data. MK designed the visual basic macro and adapted it to the simulator script. EK and GS participated in its design and coordination and helped to draft the manuscript. All authors read and approved the final manuscript.

## Supplementary Material

Additional file 1 **Standardized Resuscitation Scenario Script**. the file contains the scenario script and can be imported with the simulator software. A direct import by the MÜSE software is not supported. However, translation of the script into this platform should be possible but was not tested by the authors. In such a case, the macro (file 2) has to be adapted to the MÜSE log files Click here for file

Additional file 2 **Logfile-Macro**. the file contains a visual-basic macro that extracts the performance variables from the log files that are produced during the scenaios by the simulator software. It is obligatory to use the CPRscript for the resuscitation scenarios. Click here for file

## References

[B1] Boulet JR Murray MD Simulation-based Assessment in Anaesthesiology Anesthesiology 2010 112 1041 52 10.1097/ALN.0b013e3181cea265 20234313

[B2] Brennan RT Braslow A Batcheller AM Kaye W A reliable and valid method for evaluating cardiopulmonary resuscitation training outcomes Resuscitation 1996 85 85 93 10.1016/0300-9572(96)00967-7 8896048

[B3] Scavone BM Sproviero MT McCarthy RJWCA Sullivan JT Siddall VJWLD Development of an Objective Scoring System for Measurement of Resident Performance on the Humans Patient Simulator Anesthesiology 2006 105 260 6 10.1097/00000542-200608000-00008 16871059

[B4] Perkins GD Hulme J Tweed MJ Variability in the assessment of advanced life support skills Resuscitation 2001 50 281 6 10.1016/S0300-9572(01)00434-8 11719157

[B5] Wayne DB Butter J Siddall VJ Fudala MJ Wade LD Feinglass J McGaghie WC Mastery Learning of Advanced Cardiac Life Support Skills by Internal Medicine Residents Using Simulation Technology and Deliberate Practice J Gen Intern Med 2006 21 251 6 10.1111/j.1525-1497.2006.00341.x 16637824 PMC1828088

[B6] Ringsted C Lippert F Hesselfeldt R Rasmussen MB Mogensen SS Frost T Jensen ML Jensen MK Van der Vleuten C Assessment of Advanced Life Support competence when combining different test methods - Reliability and validity Resuscitation 2007 75 153 60 10.1016/j.resuscitation.2007.03.003 17467869

[B7] Brett-Fleegler MB Vinci RJ Weiner DLHSK Shih M Kleinman ME A simulator-based tool that assesses pediatric resident resuscitation competency Pediatrics 2008 121 e597 e603 10.1542/peds.2005-1259 18283069

[B8] Wayne DB Butter J Siddall VJ Fudala MJ Linquist LA Feinglass J Wade LD McGaghie WC Simulation-based Training of Internal Medicine Residents in Advanced Cardiac Life Support Protocols: A Randomized Trial Teach Learn Med 2005 17 210 6 16042514 10.1207/s15328015tlm1703_3

[B9] Wayne DB Didwania A Feinglass J Fudala MJ Barsuk JH McGaghie WC Simulation-based education improves quality of care during cardiac arrest team responses at an academic teaching hospital Chest 2008 133 56 61 10.1378/chest.07-0131 17573509

[B10] Wayne DB Siddall VJ Butter J Fudala MJ Wade LD Feinglass J McGaghie WC A longitudinal study of internal medicine residents' retention of advanced cardiac life support skills Acad Med 2006 81 S9 S12 10.1097/00001888-200610001-00004 17001145

[B11] Perkins GD Simulation in resuscitation training Resuscitation 2007 73 202 11 10.1016/j.resuscitation.2007.01.005 17379380

[B12] Sahu S Lata I Simulation in resuscitation teaching and training, an evidence based practice review J Emerg Trauma Shock 2010 3 378 84 10.4103/0974-2700.70758 21063561 PMC2966571

[B13] Lo B Devine A Evans D Byars DV Lamm OY Lee R Comparison of traditional versus high-fidelity simulation in the retention of ACLS knowledge Resuscitation 2011 82 1440 3 10.1016/j.resuscitation.2011.06.017 21764498

[B14] Deakin C Nolan J Soar J Sunde K Koster RW Smith GB Perkins GD European Resuscitation Council Guidelines for Resuscitation 2010. Section 4. Adult advanced life support Resuscitation 2010 10 1305 52 10.1016/j.resuscitation.2010.08.017 20956049

[B15] Berden H Pijls N Willems FF Hendrick J Crul JF A scoring system for basic cardiac life support skills in training situations Resuscitation 1992 23 21 31 10.1016/0300-9572(92)90159-A 1315067

[B16] Thoren AB Axelsson A Holmberg S Herlitz J Measurement of skills in cardiopulmonary resuscitation - do professionals follow given guidelines? Eur J Emerg Med 2001 8 169 76 10.1097/00063110-200109000-00002 11587460

[B17] Kramer-Johansen J Edelson D Losert H Kohler K Abella B Uniform reporting of measured quality of cardiopulmonary resuscitation (CPR) Resuscitation 2007 74 406 17 eng 10.1016/j.resuscitation.2007.01.024 17391831

[B18] Abella BS Edelson DP Kim S Retzer E Myklebust H Barry AM O'Hearn N Hoek TL Becker LB CPR quality improvement during in-hospital cardiac arrest using a real-time audiovisual feedback system Resuscitation 2007 73 54 61 10.1016/j.resuscitation.2006.10.027 17258853

[B19] Perkins GD Boyle W Bridgestock H Davies S Oliver Z Bradburn S Green C Davies RP Cooke MW Quality of CPR during advanced resuscitation training Resuscitation 2008 77 69 74 10.1016/j.resuscitation.2007.10.012 18083288

